# Characteristics of Patients With Alpha-1 Antitrypsin Deficiency From Rural Appalachia: A Retrospective Single-Center Study

**DOI:** 10.7759/cureus.56395

**Published:** 2024-03-18

**Authors:** Sandhya Kolagatla, Dedeepya Gullapalli, Avinash Vangara, Regina Chan, Derek Jernigan, Nagabhishek Moka, Subramanya Shyam Ganti

**Affiliations:** 1 Internal Medicine, Appalachian Regional Healthcare, Whitesburg, USA; 2 Internal Medicine, Appalachian Regional Healthcare, Harlan, USA; 3 Physical Medicine and Rehabilitation, Albany Medical College, Albany, USA; 4 Internal Medicine, Kettering Medical Center, Kettering, USA; 5 Oncology, Hematology, and Internal Medicine, Appalachian Regional Healthcare, Hazard, USA; 6 Internal Medicine/Pulmonary Critical Care, Appalachian Regional Healthcare, Harlan, USA

**Keywords:** alpha-1 antitrypsin, emphysema, copd, panniculitis, serpina

## Abstract

Alpha-1 antitrypsin (AAT) deficiency, an autosomal co-dominant inherited condition, significantly impacts lung and liver functions, with mutations in the SERPINA1 gene, notably the Z allele, playing a pivotal role in disease susceptibility. This retrospective descriptive study from a rural Eastern Kentucky pulmonary clinic aimed to characterize patients with AAT deficiency, focusing on demographic, clinical, and laboratory parameters extracted from electronic health records (EHR) of Appalachian Regional Healthcare (ARH). Among 100 patient encounters, 56 were analyzed, revealing notable sex-based differences in smoking rates and co-existing conditions, with males showing higher rates of black lung and chronic obstructive pulmonary disease. In comparison, females exhibited higher rates of asthma, COVID-19, pneumothorax, and obstructive sleep apnea. The study emphasizes the importance of understanding genotype-phenotype correlations and demographic factors in assessing AAT deficiency, advocating for further research to refine management strategies and elucidate causal relationships.

## Introduction

Alpha-1 antitrypsin (AAT) deficiency is inherited in an autosomal co-dominant fashion, which means two different alleles are expressed, one from each parent. The most common gene involved in AAT is SERPINA1 [1]. AAT is a protease inhibitor of elastase, a proteolytic enzyme. The homozygous and heterozygous genotypes involved with AAT deficiency are MS, MF, MZ, SS, SZ, ZZ, and null. The genotypes determine the risk of developing emphysema and other organ involvement. Most commonly, AAT deficiency affects the lungs, liver, and rarely the skin [2].

This article was previously presented as a meeting abstract at the 2023 CHEST Annual Meeting on October 9, 2023.

## Materials and methods

This retrospective descriptive study was conducted on patients with AAT deficiency. This study was approved by the Appalachian Regional Healthcare Institutional Review Board (IRB). As per IRB requirements, written consent was waived for this project, as it is a retrospective study, which includes the abstraction of data from medical records.

All the patients were from a single pulmonary clinic, and the data were extracted from the electronic health records (EHR) of the Appalachian Regional Healthcare (ARH) clinic allocated in rural Eastern Kentucky. The information for all patients, including demographic data, clinical characteristics, and laboratory parameters, was extracted electronically.

## Results

The study used descriptive analysis to summarize the characteristics of patients with AAT deficiency. Data for 100 patient encounters with AAT deficiency were extracted from EHR, and 44 patients were excluded due to missing variables. We conducted the preliminary analysis on the remaining 56 patient encounters. The characteristics studied were age, BMI, smoking, lung disease, pulmonary function, genotype, and alpha-1 level and were stratified by sex. Table 1 demonstrates the characteristics of patients with AAT deficiency, stratified by sex.

**Table 1:** Characteristics of patients with alpha-1 antitrypsin deficiency, stratified by sex. CAD: coronary artery disease; COPD: chronic obstructive pulmonary disease; OSA: obstructive sleep apnea.

Characteristics	Male	Female
	n = 27	n = 29
Age		
Mean (SD)	59.3 (12.1)	65.4 (13.3)
Median (IQR)	62 (57-64)	67 (57-74)
Age group, n (%)		
<40 years	2 (7.4)	2 (6.9)
41 to 50 years	1 (3.7)	2 (6.9)
51 to 60 years	6 (22.2)	9 (31.0)
61 to 70 years	9 (33.3)	9 (31.0)
>70 years	11 (40.7)	5 (17.2)
BMI (kg m2)		
Mean	28.1 (5.9)	29.8 (11.4)
Median (IQR)	29.9 (23.7-31.0)	29.3 (22.8-32.5)
BMI categories, n (%)		
<18.5 (underweight)	1 (3.7)	4 (13.8)
18.5 to 24.9 (normal or healthy)	9 (33.3)	6 (20.7)
25.0 to 29.9 (overweight)	5 (18.5)	5 (17.2)
>30.0 (obese)	14 (51.9)	12 (41.4)
Smoking		
Active	20 (74.1)	17 (58.6)
Non-smoker	7 (25.9)	12 (41.4)
Respiratory/lung disease, n (%)		
Asthma, n (%)	3 (11.1)	8 (27.6)
Black lung, n (%)	11 (40.7)	0
CAD, n (%)	7 (25.9)	6 (20.7)
COVID, n (%)	1 (3.7)	4 (13.8)
Pneumothorax, n (%)	8 (29.6)	13 (44.8)
Any malignancy in the past, n (%)	1 (3.7)	3 (10.3)
COPD, n (%)	22 (81.5)	16 (55.2)
OSA, n (%)	8 (29.6)	10 (34.5)
Pulmonary function, n (%)		
Forced expiratory volume 1/forced vital capacity, mean (SD)	63.1 (11.9)	69.4 (14.3)
Genotype, n (%)		
MS	15 (55.6)	19 (65.5)
MZ	12 (44.4)	10 (34.5)
Alpha 1 level, n (%)		
High	2 (7.4)	0
Low	5 (18.5)	1 (3.4)
Normal	20 (74.1)	28 (96.6)

The results reveal a higher prevalence of AAT deficiency among males under 40 years and over 70 years than females in these age groups. Additionally, individuals with a BMI above 30 and those who smoke are more likely to have AAT deficiency. Females with co-existing conditions such as asthma, COVID-19, pneumothorax, and obstructive sleep apnea exhibit a higher incidence of AAT deficiency compared to males with these conditions. Conversely, males have a higher prevalence of AAT deficiency when co-existing with conditions like black lung, coronary artery disease, and chronic obstructive pulmonary disease (COPD). The MS genotype is more common among males and females with AAT deficiency, followed by the MZ phenotype. Moreover, alpha-1 levels are generally higher in males, but a significant proportion of both genders with AAT deficiency exhibit normal alpha-1 levels.

## Discussion

AAT deficiency is inherited in an autosomal co-dominant fashion, which means two different alleles are expressed, one from each parent. SERPINA1 gene located on chromosome 14 is the common gene involved in AAT deficiency. There are many variant alleles of approximately 150 associated with AAT deficiency. The normal allele is referred to as "M." The critical variant is the "Z" allele that causes AAT deficiency by homozygous substitution of glutamic acid by lysine at position 342 (Glu342Lys) [3]. AAT is a protease inhibitor, denoted as "PI" [4]. The alleles are denoted with related letters. "PI*MM" refers to a protease inhibitor homozygosity for the normal gene. "PI*ZZ" refers to a protease inhibitor homozygosity for the Z allele. "PI SS" refers to a protease inhibitor homozygosity for the S allele. "PI*SZ" refers to protease inhibitor heterozygosity for S and Z alleles. "S" mutation is due to the substitution of glutamic acid by valine at position 264. PI*SZ are at increased risk of lung pathology in smokers, whereas PI*SS are not at increased risk [5].

Hepatocytes secrete AAT, and its action is to protect lung elasticity by inhibiting neutrophil elastase. Gain of function point mutation leads to liver disease by accumulation of misfolded AAT. The first three exons, named 1A, 1B, and 1C, are untranslated regions (UTR). The last four exons have the coding region and the terminal UTR [6].

The clinical manifestations can either be limited to the lungs and liver or can be systemic and involve the skin and sometimes blood vessels.

Pulmonary symptoms can range from cough to respiratory insufficiency. AAT deficiency patients, even with no to minimal smoking, will have panacinar emphysema affecting the lower lobes [3], and sometimes, it can affect the upper lobes as well. Emphysema is caused by loss of function mutation, which means there is an imbalance between neutrophil elastase in the lung, which destroys elastin, and the elastase inhibitor AAT, which is synthesized in hepatocytes and protects against proteolytic degradation of elastin [6].

Lung inflammation and destruction are caused by the chemotactic nature of Z antitrypsin, along with cigarette smoking. The progression of pulmonary function status is based on the frequency of COPD exacerbations. We can diagnose it with AAT level, computed tomography (CT) of the chest, pulmonary function tests (PFTs), and diffusion capacity of the lung measurement [7]. COPD in AAT will be treated like standard COPD with long-acting beta-2 agonists (LABA), long-acting muscarinic antagonists (LAMA), and or inhaled corticosteroids (ICS). Combination therapy of LABA with ICS has no statistical significance on all-cause mortality in COPD patients [8].

Hepatic manifestations can vary from neonatal hepatitis to hepatocellular carcinoma. The symptoms are caused by accumulating unsecreted AAT molecules due to the gain of function mutation. Diagnosis can be made by measuring the levels of aspartate aminotransferase, alanine aminotransferase, bilirubin, albumin, prothrombin time, and alpha-fetoprotein, liver ultrasound, and CT scan of the liver [9].

The skin manifestations are commonly seen in the PI*ZZ phenotype, and they can present with painful subcutaneous nodules known as panniculitis due to the accumulation of neutrophils causing inflammation.

The other systemic manifestations include aneurysms of arteries and great blood vessels [7]. Painful cutaneous panniculitis is diagnosed by histology of the biopsied specimen, which shows necrosis, acanthosis, and neutrophil accumulation. According to Lopes et al., there is no standard treatment protocol for panniculitis, but we can try steroids, colchicine, and dapsone [10].

An analysis of the German registry demonstrated that female patients with GOLD I-IV showed significantly lower numbers of pack-years and BMI. Further, this study revealed a significant delay in diagnosis for female patients [11]. Another study by Xue et al. demonstrated high levels of correlation between obesity and AAT levels [12]. Another observational study of the international registry demonstrated COPD in 57.2% and bronchiectasis in 22% of patients [13].

Limitations

This study has several limitations. Recall bias and database documentation errors are derived from the intrinsic study design. Along with these limitations, we could not complete a multivariate analysis because of the small sample size due to several missing variables. Racial characterizations for patients were not captured in the datasets for the study. Thus, racial disparities related to the AAT could not be measured. Dates were based on the dates of office appointments to the ARH system, not the date of symptom onset or diagnosis. Our team could also not assess the differences in severity or stage of comorbid conditions because only the International Classification of Diseases, Tenth Revision (ICD-10) master codes were utilized.

Future studies comparing the clinical impacts of specific variants in rural Appalachian and urban or suburban communities should be assessed to unveil potential health disparities between the communities. Further, retrospective or prospective studies examining the effects of different therapeutic modalities, such as intravenous augmentation therapies, in this particularly comorbid patient population may provide interesting insight.

## Conclusions

This descriptive study elucidates the demographic and clinical characteristics of patients with AAT deficiency. Elderly and high BMI individuals are more commonly affected. Among the 100 patient encounters, notable sex-based differences in smoking rates and co-existing conditions were observed, with males showing higher rates of black lung and COPD, while females exhibited higher rates of asthma, COVID-19, pneumothorax, and obstructive sleep apnea. The predominant genotype distribution comprised MS and MZ types, with males demonstrating higher alpha-1 levels than females. However, causative analyses were limited by sample size constraints. These findings underscore the imperative for further research endeavors aimed at refining management strategies and elucidating causal relationships, thereby enhancing the understanding and management of AAT deficiency.
